# Thermal Behavior and Mechanical Properties of Different Lattice Structures Fabricated Using Selective Laser Melting

**DOI:** 10.3390/ma17225603

**Published:** 2024-11-16

**Authors:** Hui Liu, Gaoshen Cai, Kai Peng, Haozhe Jin, Antonov Alexander

**Affiliations:** 1 School of Mechanical Engineering, Zhejiang Sci-Tech University, Hangzhou 310018, China; 2 Key Laboratory of Advanced Manufacturing Technology of Zhejiang Province, School of Mechanical Engineering, Zhejiang University, Hangzhou 310027, China; 3 Institute of Flow-Induced Corrosion, Zhejiang Sci-Tech University, Hangzhou 310018, China; 4 Innovative Technologies of Mechanical Engineering Faculty, Yanka Kupala State University of Grodno, 230023 Grodno, Belarus

**Keywords:** selective laser melting, residual stress, numerical simulation, lattice structure, mechanical property

## Abstract

In this study, the size of molten pool and the porosity of parts under different processing parameters are studied using numerical simulation. According to the results, the appropriate processing parameters were selected to simulate the temperature and residual stress distribution during the forming process of body-centered cube (BCC), face-centered cube (FCC) and rhombic dodecahedron (Dode) lattice structures. In addition, three lattice structures were fabricated via selective laser melting (SLM) technology, and quasi-static compression experiments were carried out to study their mechanical properties. The results show that the high temperature parts of the three structures are all under the node and their adjacent pillars, and the closer to the nodes, the higher the temperature. The residual stress of the Dode structure is the highest, reaching 1218.2 MPa. It is also found that the residual stress in the Z direction is the largest, which plays a dominant role in the forming process. Through compression experiments, it is found that diagonal shear failure occurs in all three lattice structures, and Dode shows the best compression performance.

## 1. Introduction

Titanium alloys have the advantages of low density, corrosion resistance, excellent fatigue strength and heat resistance [1]. Due to these characteristics, titanium alloys have great potential as lightweight structural materials. The excellent properties of Ti6Al4V make it widely used, such as gas turbines, automotive parts and medical implants and prostheses, which are welcomed by many industries [2,3].

A lattice structure has the advantages of light weight, high specific strength and great heat dissipation. It has attracted much attention in aerospace, biomedical and other fields [4,5,6]. The traditional manufacturing methods of a lattice structure include investment casting [7], snap fit, expanded metal sheet, metallic wire assembly [8], etc. However, it is difficult to manufacture geometrically complex parts using traditional manufacturing techniques. Additive manufacturing technology can overcome this difficulty and realize the manufacture of complex structures.

Selective laser melting (SLM) is a common metal additive manufacturing technology [9,10]. SLM technology uses a laser as a heat source to selectively melt the powder according to the shape of the model, and the powder is repeatedly melted until the part is manufactured [11,12]. Although the preparation of lattice structures using SLM technology has a good prospect, defects will occur due to the limitations of the process itself [13]. In addition, the powder particles undergo two processes of rapid melting and rapid solidification in the SLM forming process, resulting in large thermal stress and temperature difference, resulting in large residual stress and warpage deformation, affecting the accuracy and mechanical properties [14,15]. In order to improve the forming quality, it is necessary to study the residual stress.

Zhao et al. [16] studied the influence of different processing parameters on the residual stress of SLM-formed parts. It is found that the size and temperature gradient of molten pool are affected by the change of processing parameters. The larger the temperature gradient of the molten pool, the larger the residual stress, and the maximum residual stress there is in the overlapping area of the molten pool. The research of Wang et al. [17] showed that laser power, scanning speed and scanning strategy have different effects on the residual stress of parts, and the distribution of residual stress can be controlled by changing the processing parameters. In addition, it has also been found that there is a heat accumulation effect in the process of SLM manufacturing parts, and the existence of the heat accumulation effect makes the residual stress increase. Gan et al. [18] found that the angle and diameter of the lattice structure pillar significantly affect the residual stress. The residual stress can be reduced by increasing the inclination angle and decreasing the diameter of the pillar. The change of pillar length has little effect on the residual stress.

The measurement methods of residual stress include the drilling method, neutron diffraction method, X-ray method, etc. [19]. The drilling method requires holes to be drilled on the surface of the component, which is difficult to achieve for a lattice structure. Tobias et al. [20] introduced a non-destructive neutron diffraction method to measure the residual stress in lattice structures. However, they proposed that due to the particularity of the lattice structure, it is difficult to obtain accurate results and prone to errors.

At present, there are relatively few studies on the thermal behavior of lattice structures. Therefore, several types of lattice structures such as BCC, FCC and Dode were selected in this study, and the temperature change and residual stress distribution of the Ti6Al4V lattice structure were systematically studied. The parts were prepared using SLM technology, and their compression response and failure behavior were studied.

## 2. Materials and Methods

### 2.1. Materials and Manufacturing

**Figure 1:**
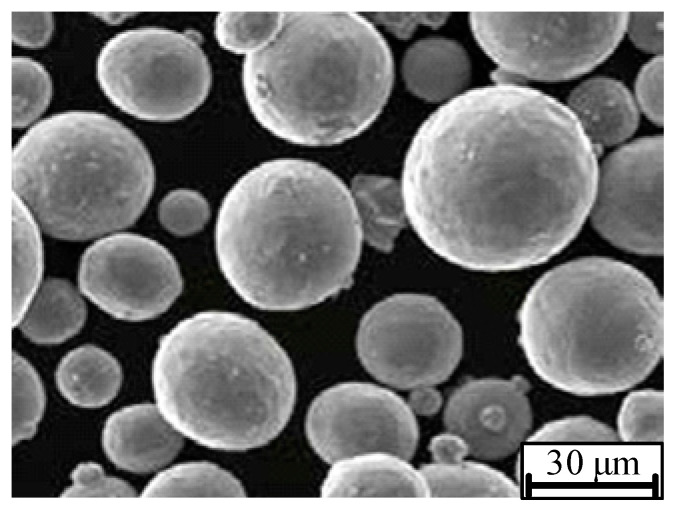
SEM image of Ti6Al4V powder.

The raw material is Ti6Al4V powder produced by Bright Laser Technologies Co., Ltd. (Xi’an, China). The chemical composition of the powder is listed in Table 1. JSM-5610LV scanning electron microscopy (SEM) images show that the morphology of the observed powder particles is close to spherical, as shown in Figure 1.

**Figure 2:**
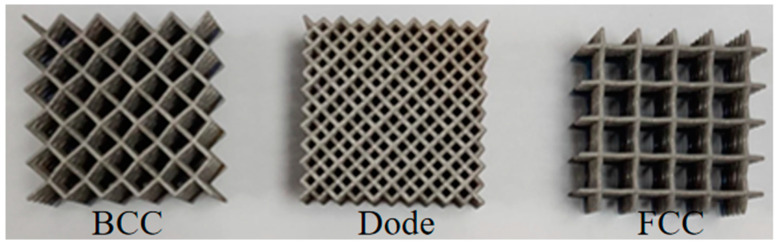
Lattice structure fabricated using SLM.

The lattice structures were fabricated using the company’s BLT-A300 + 3D printer using laser power of 300 W, scanning speed of 1000 mm/s, scanning spacing of 0.1 mm and layer thickness of 0.03 mm, as shown in Figure 2.

In order to test the mechanical properties of the lattice structures, a quasi-static compression experiment was carried out using a microcomputer-controlled electronic universal testing machine (manufactured by Ji’nan Tenson Testing Machine Manufacturing Co., Ltd., Jinan, China) with a maximum load of 100 kN, and the compression was carried out at a speed of 1 mm/min until the part failed. Then, the fracture morphology was observed via SEM, and the fracture mechanism of the samples were analyzed.

### 2.2. Numerical Simulation

The thermal behavior of SLM is very complex. To obtain accurate simulation results, it is necessary to have the temperature control equation, heat source model, initial conditions and boundary conditions. In this study, an Nd: YAG fiber laser was used for SLM, and the laser energy density was approximately Gaussian distribution. The Gaussian heat source function is shown in Equation (1) [21]:

(1) $$q=\frac{2AP}{\pi R_{L}^{2}}\exp(-\frac{2r^{2}}{R_{L}^{2}})$$

where *A* is the laser absorption rate of the powder, *P* is the laser power, *R_L_* is the radius of the laser spot, and *r* is the radial distance from the node to the center of the laser spot.

In addition, the correct initial conditions and boundary conditions should be set. Equation (2) is the initial condition.

(2) $$T{|}_{t=0}=T_{0}\left(x,y,z,t\right)$$

where *T* is the ambient temperature.

The boundary conditions in the numerical simulation model can be expressed using Equation (3) and (4), respectively [22]:

(3) $$-\lambda_{e}\frac{\partial T}{\partial n}=h(T-T_{m})$$

(4) $$-\lambda_{e}\frac{\partial T}{\partial n}=\sigma_{SB}\psi (T^{2}+T_{m}^{2})(T+T_{m})(T-T_{m})=\beta_{0}(T-T_{m})$$

where *λ_e_* is the effective thermal conductivity of the powder, n is the vector of the top of the powder bed along the normal direction, h is the convective heat transfer coefficient, *T_m_* is the ambient temperature, *σ_SB_* is the Stefan-Boltzman constant, *ψ* is the reflectivity of the material, and *β*_0_ is the thermal radiation coefficient.

The influence of temperature change on the thermal physical property parameters of Ti6Al4V material used in the numerical simulation is shown in Table 2 [23].

**Figure 3:**
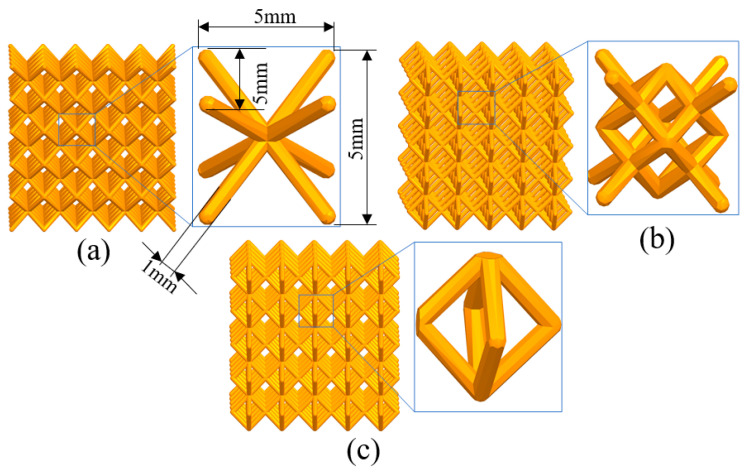
Lattice structures: (**a**) BCC, (**b**) Dode and (**c**) FCC.

NX was used to model the lattice structure, as shown in Figure 3; the size of each unit was 5 × 5 × 5, and the diameter of the pillar is 1 mm. The model was imported into ANSYS Workbench for simulation. The material is Ti6Al4V powder. The processing parameters are the same as the actual manufacturing. Tetrahedral mesh with a unit length of 0.5 mm was used.

## 3. Optimization of SLM Processing Parameters

### 3.1. The Influence of Processing Parameters on the Molten Pool

**Figure 4:**
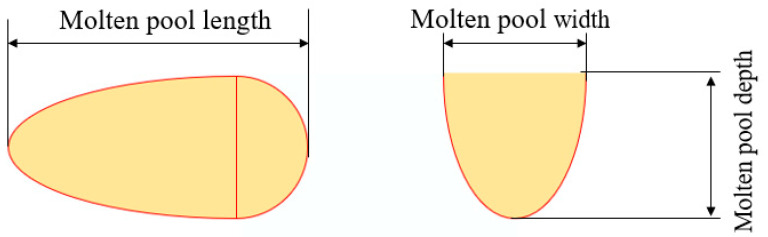
Molten pool shape.

The schematic diagram of the molten pool shape is shown in Figure 4. The shape of the molten pool is greatly affected by the process parameters, which are mainly reflected in the width and depth of the molten pool. Therefore, the same method as the previous study was used to study the influence of process parameters on the molten pool geometry [24,25].

**Figure 5:**
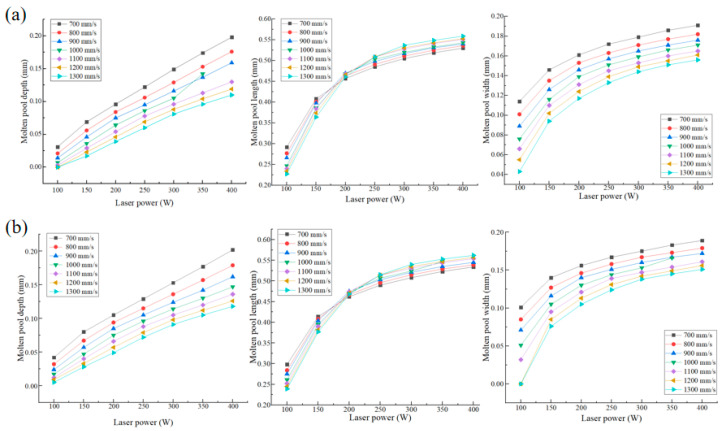
Molten pool size of different processing parameters: (**a**) 0.03 mm layer thickness and (**b**) 0.04 mm layer thickness.

Through the combination of different processing parameters, a variety of combinations are obtained. The influence of processing parameters on the molten pool is shown in Figure 5. With the increase of laser power, the depth, width and length of the molten pool increase. With the increase of scanning speed, the depth and width of the molten pool decrease, and the length of the molten pool decreases first and then increases. In the study of Chen [26] and Zhao et al. [27], the influence of process parameters on the size of the molten pool is consistent with the rule in this study.

**Figure 6:**
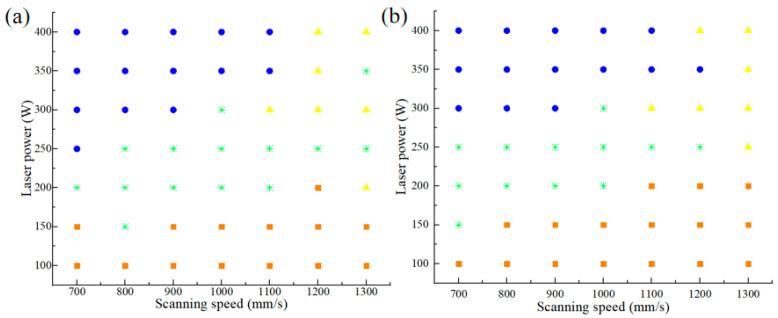
Molten pool quality of different processing parameters: (**a**) 0.03 mm layer thickness and (**b**) 0.04 mm layer thickness.

The remelting densification mechanism is proposed as a method to eliminate residual pores. When the current powder layer is melted, sufficient energy input allows the previous layer of powder to melt again, which is beneficial to reduce the porosity of the previous layer [28]. Therefore, in order to obtain a good molten pool, at least a depth of 2.5 layers of molten pool is required, because the penetration depth of 2.5 layers can reduce the porosity by remelting the previous layer. The molten pool morphology of different process parameters is shown in Figure 6. Different patterns represent different weld pool sizes. The orange square indicates that the depth of the molten pool is too shallow, the blue dots indicate that the molten pool is too deep, the yellow triangle indicates that the molten pool is too long, and the green stars indicate that the size of the molten pool is reasonable. Compared with 0.04 mm layer thickness, 0.03 mm layer thickness can produce a more suitable molten pool size.

### 3.2. The Influence of Processing Parameters on Porosity

**Figure 7:**
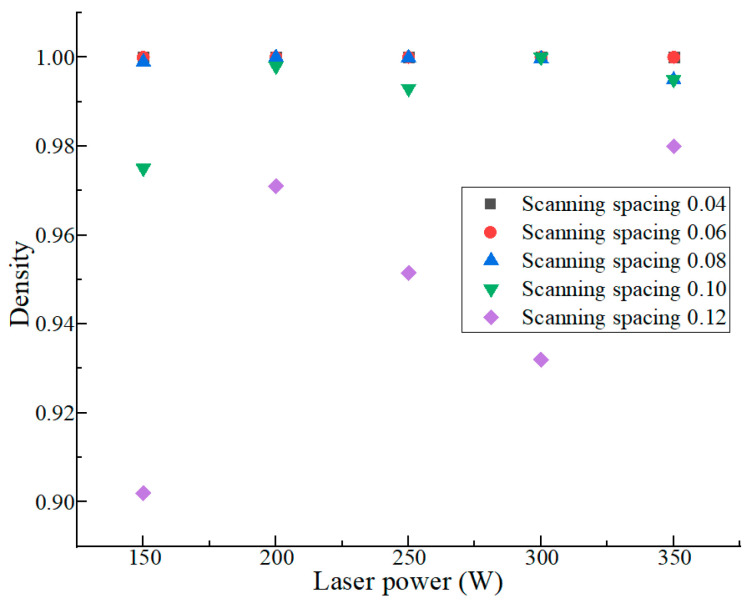
The simulation results of porosity.

According to the simulation results of the molten pool, the combination of the processing parameters in the green part of Figure 6 was selected for porosity simulation. In addition, the new variable scan spacing was added. There are many reasons that affect the porosity. In the manufacturing process, the entrained gas in the powder may remain in the part. Contaminant degassing may occur during the formation of the molten pool. During the solidification process, a small amount of gas vapor may also be retained in the molten pool, forming pores inside the part. The results are shown in Figure 7; when the scanning spacing does not exceed 0.1 mm and the laser power is 300 W, the porosity is low.

Considering the simulation results of molten pool and porosity, the optimized process parameters are as follows: laser power 300 W, scanning speed 1000 mm/s, layer thickness 30 μm, scanning spacing 0.1 mm.

## 4. Thermal Behavior

### 4.1. Temperature Distribution of Different Cell Lattice Structures

**Figure 8:**
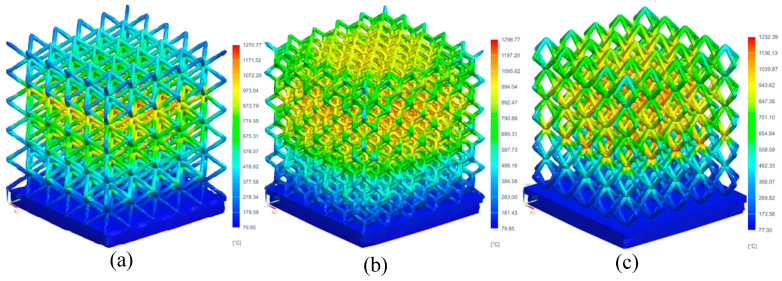
Temperature distribution of different cell lattice structures: (**a**) BCC, (**b**) Dode and (**c**) FCC.

The temperature change and residual stress of the three lattice structures were simulated using the optimized process parameters. Figure 8 shows the temperature distribution of three different cell structures during SLM forming. The high temperature parts of the three structures are all under the nodes and their adjacent pillars. The closer to the node, the higher the temperature. This is due to the repeated remelting near the node, and the continuous accumulation of temperature making the temperature rise. The maximum temperatures of BCC, Dode and FCC are 1270.77 °C, 1298.77 °C and 1232.39 °C, respectively. Among the three structures, the Dode structure has the highest temperature, mainly because the Dode structure is complex, the number of pillars is more than that of other structures, and the total input of energy in the manufacturing process is high and the heat dissipation is poor, making its highest temperature higher than the other two structures.

### 4.2. Residual Stress of Different Cell Lattice Structures

**Figure 9:**
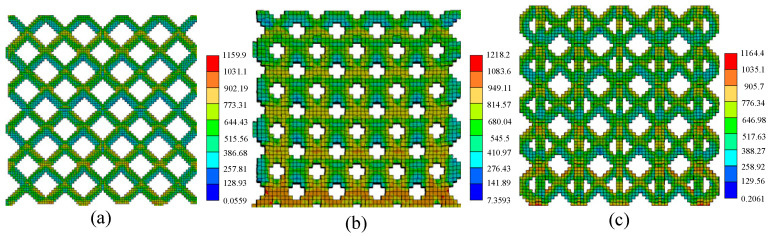
Residual stress distribution of different cell lattice structures: (**a**) BCC, (**b**) Dode and (**c**) FCC.

Figure 9 is the residual stress distribution map of different cell lattice structures. It can be found that among the three structures, the equivalent residual stress of the Dode structure is the largest, reaching 1218.2 MPa. The stress of the FCC structure reached 1164.4 MPa, and the stress of the BCC structure reached 1159.9 MPa. The complexity of the Dode structure makes the internal heat dissipation poor, so that it has the greatest residual stress. The maximum residual stress of each structure is at the node, which is because the temperature at the node is higher than at other parts in the forming process (Figure 8), and there is a large temperature gradient.

**Figure 10:**
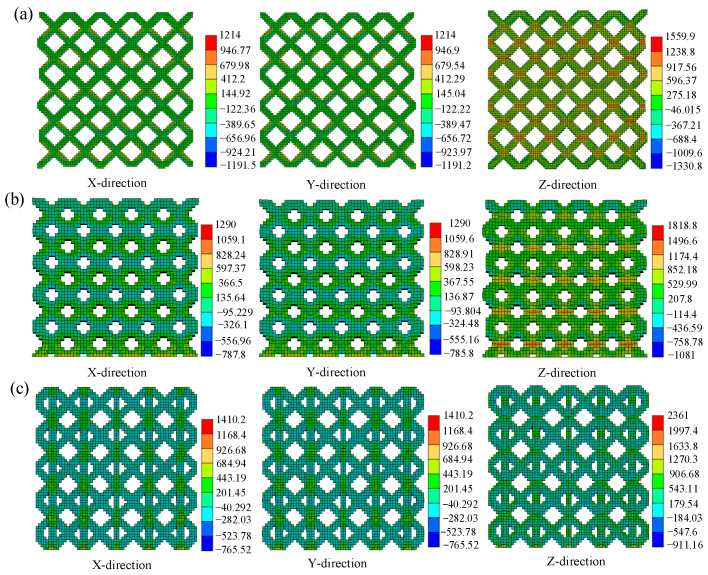
Residual stress distribution in different directions: (**a**) BCC, (**b**) Dode and (**c**) FCC.

The stress in different directions was extracted and the stress distribution of the lattice structure was further analyzed. The distribution of residual stress in BCC, Dode and FCC structures is shown in Figure 10. The equivalent residual stress component in Z direction is the largest, and the stress magnitude in other directions is similar. This is due to the excessive temperature gradient at the connection between the substrate and the component. The maximum tensile stresses of BCC, Dode and FCC in the Z direction are 1559.9 MPa, 1818.8 MPa and 2361 MPa, respectively. The maximum compressive stresses are 1330.8 MPa, 1081.0 MPa and 911.16 MPa, respectively.

## 5. Results and Discussion

### 5.1. Quasi-Static Compression Experiment

**Figure 11:**
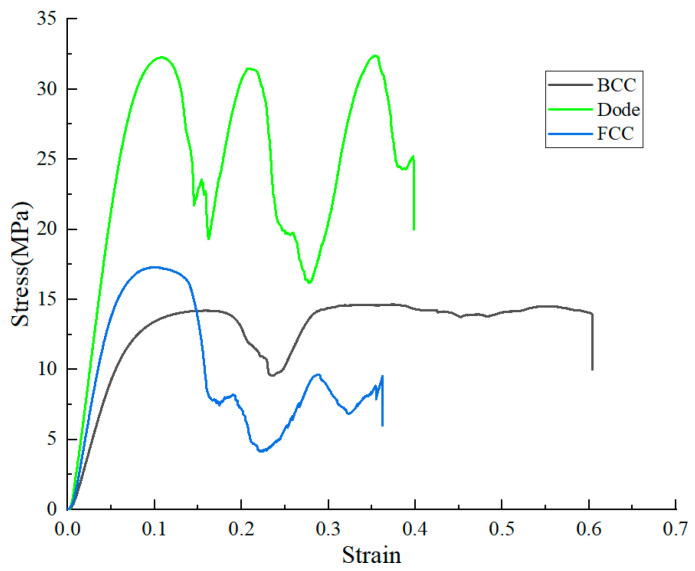
Stress–strain curve of different lattice structures.

Figure 11 shows the stress–strain curves of different lattice structures. The compression performance of different lattice structures is very different. Multiple peaks are observed in the stress–strain curve, and the final feature is that the stress drops sharply and the parts are damaged. The slope of the curve represents the elastic modulus, and the first peak stress represents the compressive strength. The slope of the Dode structure in the elastic stage is significantly larger than that of BCC and FCC. The strain at the first peak stress of all the structures is very close, but the peak stress is very different. The mechanical properties of the three lattice structures are shown in Table 3. The Dode structure has the highest elastic modulus and compressive strength, showing the best compression performance. Although FCC has a higher elastic modulus and compressive strength than BCC, it can be seen from Figure 10 that the bearing capacity decreases sharply after the first damage, and begins to be lower than the BCC structure after the strain reaches about 0.14.

### 5.2. Deformation Behavior

**Figure 12:**
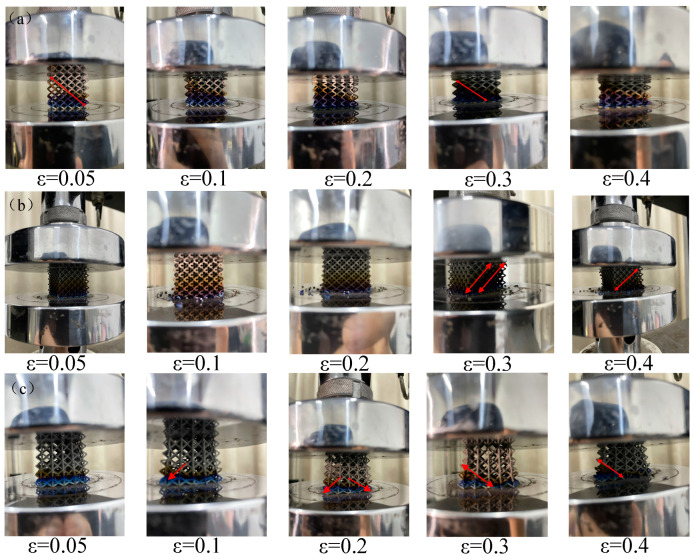
Deformation mode of lattice structure: (**a**) BCC, (**b**) Dode and (**c**) FCC.

Figure 12 shows the deformation of each lattice structure during the compression experiment. In the initial stage of the compression experiment, slight deformation occurred in each structure. As the pressure continues to be applied, the pillars of the bottom unit deform and extend outward, and the bottom deformation of the FCC structure is the most significant. They have different deformation mechanisms after the yield stage. The failure of the three structures occurs at the nodes. The pillars at the fracture of the BCC structure are compressed in a horizontal direction, and the direction of the pillars at the fracture of the FCC structure is almost unchanged. Both are caused by the bending collapse of the pillars. The relative slip of the Dode structure occurs along the 45° direction. Finally, all three structures form extrusion bands along the 45° direction, resulting in catastrophic damage. In the compression process, it is found that the compression performance of the lattice structure substrate will be affected after laser cutting. Laser cutting causes the hardness of the bottom pillar to increase, the plasticity to decrease, and the bottom pillar is more likely to break under load.

### 5.3. Fracture Morphology

**Figure 13:**
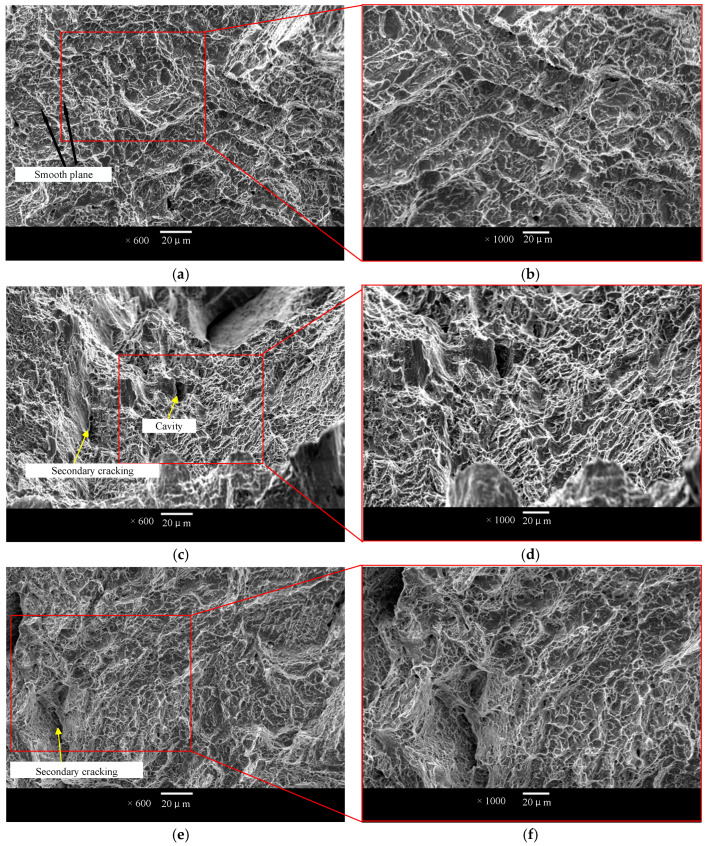
The compressive fracture morphology of BCC, Dode and FCC (**a**,**c**,**e**) and the local enlargement of the fracture morphology of the lattice structure (**b**,**d**,**f**).

Figure 13a,c,e are the compressive fracture morphology of BCC, Dode and FCC lattice structures, respectively. Figure 13b,d,f correspond to the local amplification diagram, respectively. A large number of dimples are observed in each lattice structure, which is characteristic of the ductile fracture of metals. A ductile fracture is beneficial for the structure to absorb more energy. Secondary cracks and holes are found in Figure 13c. The reason for the secondary crack is that the lattice structure tends to expand outward during the loading process of the compression experiment, resulting in high tension in the weak area in the middle of the pillar, resulting in a mixed fracture. The hole is generated by the gas residue during the manufacturing process. There are more dimples on the BCC fracture than on the FCC fracture, and the dimples on the FCC fracture surface are larger and the tearing is more obvious, indicating that the FCC nodes undergo large plastic deformation before the final fracture.

## 6. Conclusions

In this study, the processing parameters were optimized via numerical simulation. The temperature and residual stress distribution of different cell lattice structures were studied using the optimization results. The compression performance of the lattice structure was tested. The following main conclusions are drawn:

(1) The influence of different processing parameters on the molten pool and porosity were studied, and the optimization was carried out based on this. The optimized parameters were: laser power 300 W, scanning speed 1000 mm/s, powder thickness 0.03 mm and scanning spacing 0.1 mm.
(2) The thermal behavior evolution of the Ti6Al4V lattice structure in the SLM forming process was studied. The high temperature parts of the three structures are all on the lower side of the node and its adjacent pillars, and the closer to the node, the higher the temperature. The temperature of the Dode structure reaches the highest, followed by the BCC structure, and the smallest is the FCC.
(3) The node of the lattice structure is the main area of residual stress distribution, which is closely related to the high temperature distribution at the node. From the residual stress in different directions, the residual compressive stress exists at both ends of the part. The residual stress in the X and Y directions is close, and the residual stress in the Z direction has the greatest influence on the forming process. Moreover, the residual stress of the lattice structure of different cells is significantly different. The residual stress of Dode is the highest, reaching 1218.2 MPa. FCC is the second, and BCC is the lowest.
(4) The compression performance of different cell lattice structures is very different. The Dode structure has the highest elastic modulus and compressive strength, reaching 478.72 MPa and 32.25 MPa, showing the best compression performance among the three.

## Figures and Tables

**Table 1:** Chemical compositions (wt.%).

Alloying Elements	Ti	Al	V	Fe	C	N	H	O
**wt.%**	Bal.	5.5–6.75	3.5–4.5	≤0.30	≤0.08	≤0.05	≤0.015	0.08–0.13

Bal.: Balance, the amount of remaining ingredients.

**Table 2:** The mechanical property of Ti6Al4V.

Temperature (K)	Coefficient of Thermal Expansion (×10^6^/K)	Modulus of Elasticity (GPa)	The Yield Strength (MPa)	Poisson’s Ratio	Shear Modulus (MPa)
298	8.91	107	1098	0.323	0.7
589	10.12	91.8	844	0.339	2.2
700	10.82	82.4	663	0.347	2.2
811	11.31	69.6	527	0.354	1.9
923	11.71	54.7	300	0.361	1.9
1073	12.21	35.3	60	0.369	2
1123	12.37	29.3	5	0.371	2
1473	12.44	6.6	2	0.392	2
1923	12.51	0.1	0.1	0.415	0.1

**Table 3:** Mechanical properties of different lattice structures.

Sample	Elastic Modulus (MPa)	Compressive Strength (MPa)	Plateau Stress (MPa)	Yield Strength (MPa)
BCC	208.46	14.2	13.61	10.5
Dode	478.72	32.26	25.69	23.4
FCC	340.96	17.29	8.871	14.59

## Data Availability

The data presented in this study are available on request from the corresponding author.
